# Comparative pharmacokinetics of plasma- and albumin-free recombinant factor VIII in children and adults: the influence of blood sampling schedule on observed age-related differences and implications for dose tailoring

**DOI:** 10.1111/j.1538-7836.2010.03757.x

**Published:** 2010-04

**Authors:** S BJÖRKMAN, V S BLANCHETTE, K FISCHER, M OH, G SPOTTS, P SCHROTH, S FRITSCH, L PATRONE, B M EWENSTEIN, P W COLLINS, FOR THE ADVATE CLINICAL PROGRAM GROUP

**Affiliations:** * Department of Pharmaceutical Biosciences, Uppsala UniversityUppsala, Sweden; † Division of Haematology/Oncology, Hospital for Sick Children, and Department of Paediatrics, University of TorontoToronto, ON, Canada; ‡ Van Creveldkliniek, University Medical CenterUtrecht, the Netherlands; § Baxter Healthcare CorporationWestlake Village, CA, USA; ¶ Baxter Innovations GmbHVienna, Austria; ** Arthur Bloom Haemophilia Centre, Department of Haematology, Medical School of Cardiff University, University Hospital of WalesHeath Park, Cardiff, UK

**Keywords:** factor VIII, hemophilia A, pharmacokinetics

## Abstract

**Summary:**

*Background*: Dose tailoring of coagulation factors requires reliably estimated and reproducible pharmacokinetics (PK) in the individual patient. *Objectives*: To investigate the contribution of both biological and methodological factors to the observed variability of factor VIII (FVIII) PK, with the focus on differences between children and adults, and to examine the implications for dosing. *Patients*: Data from 52 1–6-year-old and 100 10–65-year-old patients with hemophilia A (FVIII ≤ 2 IU dL^−1^) in three clinical studies were included. *Results*: *In vivo* recovery was lower, weight-adjusted clearance was higher and FVIII half-life was on average shorter in children than in adults. However, a reduced blood sampling schedule for children was estimated to account for up to one half of the total observed differences. Intrapatient variance in PK was smaller than interpatient variance in 10–65-year-olds. Age and ratio of actual to ideal weight only showed weak relationships with PK parameters. Variance in PK caused large variance in the calculated dose required to maintain a target FVIII trough level during prophylactic treatment. *Conclusion*: Differences in blood sampling schedules should be taken into account when results from different PK studies are compared. However, even with this consideration, PK cannot be predicted from observable patient characteristics but must be determined for the individual. Because the influence of reducing the blood sampling was minor in comparison to the true variance between patients, a reduced blood sampling protocol can be used. Low intrapatient variability supports the use of PK measurements for dose tailoring of FVIII.

## Introduction

Prophylactic treatment of hemophilia A with factor VIII (FVIII) is based on the assumption that an adequate plasma level of exogenous coagulation factor will protect the patient from bleeding. [1–3] The plasma level (or level vs. time curve) of FVIII achieved after an infusion depends on the dose and on the patient’s pharmacokinetic (PK) response to it. Thus, regardless of whether PK calculations are performed or not, the clinician must consider interindividual variability in FVIII PK and adjust the dosing accordingly. Consequently, relationships between patient characteristics (e.g. age and body weight (BW)) and PK have been sought in order to serve as guidance to dosing. By application of PK principles, the dosage of FVIII required to reach any predetermined plasma level can then also be optimized for each individual patient [4–11].

Patient characteristics that show relationships with FVIII PK include age-dependent physiological changes [8–10,12–16] and body size and composition [15,17,18]. Thus, weight-adjusted clearance (CL) of FVIII (i.e. in mL h^−1^ per kg) has generally been found to decrease with age and/or BW during growth from infancy to adulthood, with a corresponding increase in terminal half-life (*t*_½_) [8,12,13,15,16]. However, variance in PK can arise for both true biological and technical or methodological reasons. The latter reasons for variance include differences among FVIII concentrates, methods for determining product potency and plasma FVIII levels, [7,9,18] and differences among protocol designs, especially as regards the choice of blood sampling times and the final data analysis [5,7,19–22].

The initial aim of this study was to examine relationships between FVIII PK parameters and biological characteristics, in particular differences between young children and older children/adults, using data from three prospective studies on a recombinant FVIII (rFVIII). During the evaluation, methodological issues became apparent that merited an investigation of the effects of the blood sampling schedule and repeated PK assessments in addition to the influence of patient characteristics. Consequently, this report examines contributions of both biological and technical or methodological factors to observed variability in the PK data from the three studies. Implications for blood sampling and dosing in practice are also explored.

## Methods

### Study designs

The PK data from previously treated patients (PTPs) with moderately severe to severe hemophilia A (FVIII ≤ 2 IU dL^−1^) were compiled from three clinical studies using ADVATE® rAHF-PFM [Antihemophilic Factor (recombinant), Plasma/Albumin Free Method; Baxter Healthcare Corporation, Deerfield, IL, USA]:

1. a prospective, multicenter, open-label, Phase 2/3 (pediatric) study in pediatric PTPs (1–6 years of age) with a PK evaluation [15];
2. a prospective, multicenter, Phase 2/3 (pivotal) study in PTPs ≥ 10 years of age with two randomized, double-blinded, crossover PK comparisons [23]; and
3. a prospective, multicenter, open-label, Phase 2/3 (continuation) study with a single-arm evaluation of PK after at least 75 exposure days (EDs) in only patients who completed the pivotal study.

### Patients

All patients, or their legally authorized representatives, provided written informed consent. All 52 patients (1–6 years of age) in the pediatric study were included in the PK evaluation. Of 111 patients enrolled in the pivotal study, 100 (10–65 years of age) were included in PK evaluations. Of these, 34 participated in the continuation study and were included in the PK evaluation. None of the patients had a known history of inhibitors. The characteristics of the patients are shown in Table 1.

**Table 1:** Patient age and ratio weight

	1–6-year age group (*n* = 52)					10–65-year age group (*n* = 100)				
	Min	25%	Median	75%	Max	Min	25%	Median	75%	Max
Age (years)	1.2	2.2	3.5	5.0	6.0	10.1	14.1	18.5	30.4	65.7
Weight (kg)	10.6	13.4	15.7	19.0	27.2	35.0	53.2	68.6	77.3	107.8
Height (cm)	76	91	98	111	121	135	163	172	178	191
Ratio weight*	0.77	0.96	1.04	1.11	1.54	0.71	0.97	1.05	1.20	1.69

* Ratio weight: actual/ideal weight for age. *n*, number of patients; Min, minimum of range; Max, maximum of range; 25%, 25th percentile; 75%, 75th percentile.

### Study procedures and treatment

All 52 patients in the 1–6-year age group received a single PK infusion with rAHF-PFM [15]. Of 100 patients in the 10–65-year age group, 16 received one infusion with rAHF-PFM and 83 received two infusions with biologically equivalent rAHF-PFM products, one manufactured at investigational scale and the other at commercial scale [23]. Of those patients receiving two infusions, 49 received infusions between 72 h and 30 days apart and 34 received infusions before and after at least 75 EDs.

The PK blood sampling schedule was consistent with the recommendations of the FVIII/FIX Scientific and Standardization Committee of the International Society on Thrombosis and Haemostasis (ISTH) that permit a reduced schedule for children under 5 years of age [19–22]. Doses of FVIII, infusion information and blood sampling times are given in Table 2.

**Table 2:** Pharmacokinetic study infusions and pre-infusion FVIII:C level

	1–6-year age group (*n* = 52)	10–65-year age group (*n* = 100)
Median dose (IU kg^−1^)	50 (range: 45–55)	50 (range: 40–62)
Infusion rate	Maximum of ≤ 10 mL min^−1^ over ≤ 5 min^−1^	
Total number of infusions	52†	184‡
Blood sampling schedule	Reduced schedule	Full schedule
Median preinfusion time (h)	−0.08 (−0.92 to −0.02)	−0.12 (−1.67 to 0.00)
0.25	ND	0.25 (0.17–0.40)
0.5	ND	0.50 (0.42–0.83)
1	1.0 (0.9–1.2)	1.0 (0.9–1.4)
3	ND	3.0 (2.9–4.0)
6	ND	6.0 (5.7–7.0)
9	8.1 (7.0–9.8)	9.0 (8.1–10.0)
24	24.0 (22.2–26.0)	24.0 (23.1–25.0)
28	ND	28.0 (27.0–28.5)
32	ND	32.0 (30.0–32.9)
48	48.1 (46.2–49.9)	48.1 (47.0–49.3)
Median preinfusion FVIII:C level (IU dL^−1^)	<1 (range, <1–5.9; IQR, <1–1.5)	<1 (range, <1–47.0; IQR, <1–1.4)

† Of 52 patients, each received one infusion. All infusions included all reduced sampling time points.

‡ Of 100 patients, 16 received one infusion and 84 received two infusions. One hundred and seventy-four infusions included all full sampling schedule time points; nine were missing one time point and one was missing two time points. IQR, interquartile range. *n*, number of patients. ND, not done.

### Pharmacokinetic calculations

FVIII activity assays were conducted at a central laboratory and PK parameters were essentially calculated as previously described [23]. The one-stage aPTT-based assay was used, and FVIII levels are expressed as FVIII coagulant activity (FVIII:C). Post-infusion FVIII:C levels were baseline-adjusted by the proportion of preinfusion to maximum (peak) level; that is, adjusted FVIII:C = measured FVIII:C × [1 – (pre-infusion FVIII:C/maximum FVIII:C)]. FVIII:C data from at least seven time points in the 10–65-year age group and at least three time points in the 1–6-year age group were required for subsequent PK analyses. The shape of each patient’s FVIII:C vs. time curve was evaluated by one- or two-phase linear regression [24]. The rescaled-residual sums of squares of single-phase and two-phase cases were calculated for each patient and the model with the least residual sum was chosen to yield an estimate of *t*_1/2_. At least five data points were required for a two-phase regression and the second phase was based on at least three of these. Incremental *in vivo* recovery (recovery) was calculated by a standard procedure [21] from the maximal observed plasma FVIII:C level (*C*_max_). Area under the curve (AUC) and area under the first moment curve (AUMC) were estimated by linear trapezoidal methods, with extrapolation to infinite time [7]. Weight-adjusted CL and volume of distribution at steady-state (*V*_ss_) were then calculated from dose, AUC and AUMC by standard methods [7]. All PK parameters are reported using descriptive statistics.

The impact of reducing the sampling schedule, as for the 1–6-year age group, on PK parameter estimation was evaluated by removing the 0.25, 0.5, 3, 6, 28 and 32 h postinfusion data points from the 10–65-year age group and then recalculating PK parameter values.

### Statistical analyses

Variance component analyses were performed to assess intrapatient and interpatient variance on two repeated PK measurements. Multivariate linear regression analyses were performed to investigate the association between PK parameters and patient’s characteristics. Because PK parameters were not normally distributed, values were log-transformed. The characteristics used as independent variables were age and ratio weight. The ratio weight is defined as actual weight divided by ideal weight for age and was chosen as the most suitable age-independent body mass measure. Spearman’s correlations with age were calculated for three candidate measurements: body mass index (BMI), ratio weight, and ratio BMI (= actual BMI/median BMI for age). Among these, ratio weight showed least age-dependency and was included in the final multiple regression model. Ideal weight, which is very similar to lean body mass, was estimated for patients > 18 years as 50 + 0.9 × (height in cm for each cm above 152) kg [25] and for patients ≤ 18 years by the smoothed median curves between weight and age on the NHANES data [26].

### Implications of PK estimates for dosing during prophylactic treatment

The influence of different estimates of PK parameters (i.e. from three sampling schedules: 1–6-year-olds with reduced sampling, 10–65-year-olds with full sampling, and 10–65-year-olds with reduced sampling) on the dosage of FVIII needed to maintain a 1.0 IU dL^−1^ trough level was calculated. Regular prophylactic dosing every 2 days was assumed and the minimum (or trough) level, *C*_min_, was calculated using standard one-compartment PK according to:
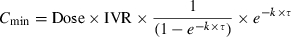


In this equation, dose is in IU kg^−1^, IVR is recovery in IU dL^−1^ per IU kg,^−1^ k is the elimination rate constant (k = ln2/*t*_*½*_), and τ is the time interval between doses (i.e. 48 h). The term 1/(1−*e*^−*k*×*τ*^) accounts for accumulation of FVIII during multiple dosing (i.e. some FVIII remaining from previous doses whenever a new dose is given). The dose required for *C*_min_ = 1.0 IU dL^−1^ was calculated using various values for recovery and *t*_½_. For biphasic FVIII:C vs. time curves, calculations were performed using the data describing the terminal phase of the curve. For the calculation of annual FVIII consumption the median body weights of 16 and 68 kg were used (for the 1–6- and 1–65-year age groups, respectively).

## Results

### Patient characteristics and PK parameter values

Baseline patient characteristics are shown in Table 1 and PK infusions are described in Table 2. No patient was excluded due to missing data points. According to the protocol, *C*_max_ was only measured at the 1-h postinfusion time point for all (52) assessments in the 1–6-year age group. For patients in the 10–65-year age group who had all 10 postinfusion samples (174/184 assessments), *C*_max_ was observed at 15 min postinfusion in 75% (130/174) of the assessments, at 30 min in 21% (36/174), at 1 h in 3.5% (6/174), and at 3 h in the remaining two assessments. Thus, in the 10–65-year age group, *C*_max_ was found at 15–30 min in the majority (166/174) of study assessments.

**Fig. 1:**
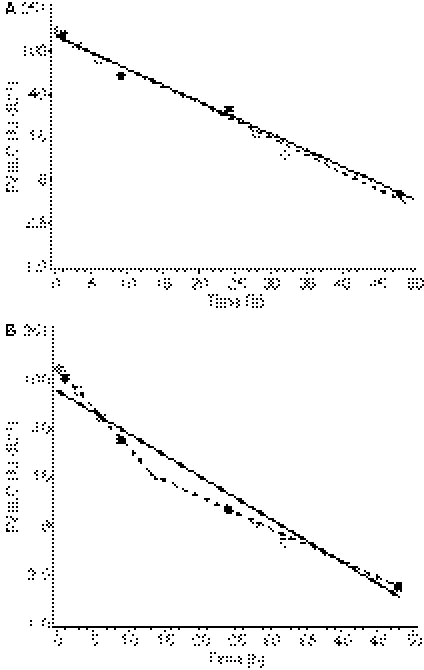
Representative FVIII:C vs. time curves from two adult patients as evaluated by one- or two-phase linear regression. When the full sampling schedule is used, the fitted curve is monophasic for patient A and biphasic for patient B. With data from the reduced sampling schedule, only monophasic curves can be fitted for both patients. Key to symbols: open and filled circles and dashed line = biphasic fit, filled circles and solid lines = monophasic fit.

According to the curve-fitting criteria (which required at least five postinfusion values for two-phase linear regression), the FVIII:C plasma level vs. time could only be described by monophasic curves in the 1–6-year age group. In contrast, in the 10–65-year age group with the full sampling schedule, the curves were better described by biphasic functions in 163/184 (89%) cases (representative curves are shown in Fig. 1). The median plasma level of exogenous FVIII:C at 48 h after the infusion was 2.5 (range: < 1.0–12) IU dL^−1^ in the 1–6-year age group and 4.4 (range: < 1.0–23) IU dL^−1^ in the 10–65-year age group. PK parameters are summarized in Table 3. Overall, patients in the older age group showed a lower median weight-adjusted CL and a longer median *t*_½_. Recoveries or *V*_ss_ could not be compared directly due to the differences in sampling times.

**Table 3:** Difference in median pharmacokinetic parameters between age groups

	Median (25th–75th percentiles) for each schedule*			Difference in medians		Median difference
	Reduced	Full	Reduced			
Parameter	1–6-year age group†	10–65-year age group‡	10–65-year age group‡	Reduced 1–6- and full 10–65-year groups	Reduced 1–6- and reduced 10–65-year groups	Reduced 10–65- and full 10–65-year groups
Maximal FVIII:C§ (IU dL^−1^)	92 (83–101)	122 (106–141)	108 (89–127)	−30	−16	−13.5
Incremental *in vivo* recovery [(IU dL^−1^)/(IU kg^−1^)]	1.84 (1.64–2.02)	2.42 (2.14–2.74)	2.16 (1.84–2.46)	−0.58	−0.32	−0.27
Clearance [mL h^−1^ kg^−1^]	4.34 (3.39–5.46)	3.26 (2.61–3.98)	3.16 (2.54–3.88)	1.08	1.18	−0.06
Volume of distribution (dL kg^−1^)	0.50 (0.45–0.58)	0.48 (0.42–0.54)	0.46 (0.39–0.52)	0.02	0.04	−0.03
Terminal half-life (h)	9.4 (8.1–10.8)	11.2 (9.6–13.4)	10.5 (9.0–12.2)	−1.8	−1.1	−0.8

See Table 2 for reduced and full sampling schedules.

† Calculated from one PK infusion for each of 52 patients, each of which included all reduced sampling time points.

‡ Calculated from one PK infusion for 49 patients and an average of two PK infusions for 51 patients, of which 10 were missing one or two time points (see Table 2).

§ Maximal concentration was observed at 15–30 min postinfusion for 96% of the assessments in the 10–65-year age group and was only measured at 1-h postinfusion in the 1–6-year age group.

### Effects of sampling schedule on estimation of PK parameters

Reducing the sampling schedule in the 10–65-year age group to mimic that in the 1–6-year age group allowed only monophasic curves to be used to describe FVIII:C vs. time. It also resulted in lower estimates (*P* < 0.001) of all five PK parameters, for example in the 10–65-year age group the median difference in *t*_½_ between the reduced and full sampling schedules was 0.80 (25th–75th percentiles: 0.38–1.5) h (Table 3). Furthermore, when differences in PK parameters were compared between the 1–6- and 10–65-year age groups, 47% of the difference in *C*_max_, 45% of the difference in recovery and 38% of the difference in *t*_½_ disappeared when the same sampling schedule was applied to both groups of patients.

### Variability in PK assessments

For all PK parameters, the proportions of intrapatient (within the same patient) variance were much smaller than the proportions of interpatient (among patients) variance between two infusions that were from 72 h to 30 days apart, as well as between those that were ≥ 75 EDs apart (Table 4). Weight-adjusted CL showed the least proportion of intrapatient variance, while *C*_max_ and recovery showed the greatest proportions.

**Table 4:** Relative proportion of variances in pharmacokinetic parameters for patients in the 10–65-year age group

	Percentage of variance between two infusions			
	3 days to 4 weeks apart (*n* = 49)		At least 75 EDs apart (*n* = 34)	
Parameter	Intrapatient (%)	Interpatient (%)	Intrapatient (%)	Interpatient (%)
Maximal FVIII:C	36	64	33	67
Incremental *in vivo* recovery	35	65	39	61
Weight-adjusted clearance	13	87	14	86
Weight-adjusted volume of distribution	24	76	21	79
Terminal half-life	22	78	35	65

### Effect of patient characteristics on PK parameters

Multivariate linear regression analyses were performed to assess associations among PK parameters and patient characteristics. The only significant correlation found in the 1–6-year age group was an increase in *t*_½_ with age (*P* = 0.01), but not with ratio weight. *t*_1/2_ was not correlated with age or ratio weight in the 10–65-year age group. However, in this group, *C*_max_ and recovery significantly increased with both age and ratio weight (*P* ≤ 0.03). In addition, weight-adjusted CL and weight-adjusted *V*_ss_ significantly decreased with age and ratio weight (*P* ≤ 0.024). All model *r*^2^ values were low, ≤ 0.31 and ≤ 0.13 in analyses for the 1–6-year and 10–65-year age groups, respectively.

### Implications for dosing during prophylactic treatment

The influence of PK estimates on calculated dose requirements that intend to maintain FVIII levels ≥ 1 IU dL^−1^ during a prophylactic treatment regimen is shown in Table 5. The recoveries were fixed at the median values for each of the three groups. Variance in *t*_½_ had a large effect on dose requirements because patients at the lower (25th) percentiles would need approximately three times as much FVIII as patients at the higher (75th) percentiles. In contrast, a decrease in recovery from the upper to the lower percentile would only require a modest proportional increase in dose (23% and 34%, respectively). Calculations using the terminal parts of the biphasic curves (compare Fig. 1) yielded almost identical results for the 10–65-year age group.

**Table 5:** Calculated dose of FVIII needed to maintain a trough level of 1.0 IU dL^−1^ during prophylactic treatment with alternate day dosing

Patient age range	Half-life (h)	Dose (IU kg^−1^)	FVIII consumption (kIU year^−1^)
1–6 years	8.1	29.7	87
	9.4	16.8	49
	10.8	10.6	31
10–65 years (full sampling)	9.6	17.9	222
	11.2	10.8	134
	13.4	6.3	79
10–65 years (reduced sampling)	9.0	17.1	213
	10.5	9.8	122
	12.2	6.3	78

## Discussion

The use of PK for any clinical or practical purpose presupposes that reported findings are reproducible and comparable, that the PK is reasonably stable in an individual and that suitable methodology is available and applied. This report examines these assumptions using FVIII PK data from a large cohort of children and adults. The median PK parameter values in this cohort were in general agreement with earlier findings for rFVIII [9,14,27–29]. In all 152 patients (1–65 years of age) there was a 5-fold interindividual variation in weight-adjusted CL (from 1.6 to 7.8 mL h^−1^ per kg) and a 4-fold variation in *t*_½_ (from 6.7 to 25 h), which gave a good range of data for investigating causes of variance.

It can be reasonably assumed that a reduced blood sampling schedule in children would influence estimates of PK parameters [20,22] as well as comparisons of PK parameters between children and adults. This assumption has to our knowledge never been tested using actual data. We found that methodological influences were as significant as the postulated biological correlates. Reducing the sampling schedule (from 10 to four postinfusion blood samples) resulted in significant changes in all estimates of PK parameters in the older children/adults. The reduced sampling schedule underestimated *C*_max_ and recovery, which can be expected because *C*_max_ occurred at 15–30 min postinfusion for 95% of the determinations with the full sampling schedule and these time points were deleted with the reduced schedule. Blood samples taken up to 30 min after the infusion yielded similar *C*_max_ values irrespective of sampling time. Therefore in clinical practice, recovery samples should be taken when convenient but within 30 min after the infusion.

Reducing the sampling schedule had only a minor impact on the estimates of CL and *V*_ss_. These parameters are based on AUC, and thus are more robust estimates compared with recovery, which is calculated from a single FVIII:C level, and also compared with *t*_½_, which depends on line fitting to various numbers of data points. In this context it must be pointed out that these findings were obtained using the one- or two-phase linear regression described by Lee *et al.* [24] and may not have been precisely the same with application of other curve-fitting methods.

Irrespective of whether the comparison was made with the calculations on the full or reduced data set from the 10–65-year age group, the recovery was lower, weight-adjusted CL was higher and *t*_½_ was shorter in the 1–6-year age group. Comparing data from the two age groups using the same sampling schedule abolished substantial proportions of the apparent difference in *C*_max_, recovery and *t*_½_. These findings are consistent with discussions in the ISTH publications [20–22] on the methodology of PK studies. For product comparison studies, blood samples should be taken at 15 (or 15 and 30) min postinfusion in addition to the 1-h sample, while for the evaluation of prophylaxis in children the reduced sampling schedule recommended by ISTH may be used. Our findings confirm that these two study designs or blood sampling schedules yield results that are not directly comparable.

Some patients had preinfusion levels exceeding 1 IU dL^−1^ FVIII:C (Table 2), which could in some cases be attributed to inadequate washout periods before administration of the study dose. However, the proportional baseline-adjustment method chosen in the data analysis (see Methods section) serves to subtract the declining FVIII:C level from a previous dose. The high preinfusion levels in some patients consequently did not affect the findings of this study.

In order to use PK parameters for individual dose optimization they must be reproducible within a patient. Intrapatient variability in recovery or *t*_½_ has been investigated and compared with interpatient variability in a few studies [16,17,23,27,30]. In the present report, similar results were obtained with short- and long-term repeated PK investigations: with 49 patients who received products that were manufactured at different facilities, using essentially identical processes and previously shown to be bioequivalent with respect to AUC and recovery, [23] within 30 days of each other and with 34 patients who received the same product before and after at least 75 EDs. In general agreement with previous findings [16,23,27,30], intrapatient variance was uniformly less than interpatient variance. *C*_max_ and recovery showed the most intrapatient variability, followed by weight-adjusted *V*_ss_ and terminal *t*_½_, while the most robust parameter, weight-adjusted CL, was highly reproducible. The reasons for these findings should be similar to those discussed for sampling reduction (above). Therefore, as previously pointed out, [7,18,21,22] CL (and by definition AUC/dose) are the best parameters to compare products while, as also discussed before [5,7,18,30], recovery is of little use for this purpose.

Within each age group, only weak relationships were found between PK parameters and the biological parameters of age and ratio weight. In the 1–6-year age group, *t*_½_ increased with age as previously described for these patients [15]. An increase in *t*_½_ may be explained either by a decrease in CL or an increase in *V*_ss_ (or a combination of both). In this data set, it appeared to be a combination of both, but the individual relationships did not reach statistical significance. In the 10–65-year age group, recovery and *C*_max_ increased, and weight-adjusted *V*_ss_ decreased, independently with age as well as with ratio weight. Importantly, all *r*^2^ values were low, which indicates that neither age nor ratio weight can be used as predictors of PK parameters in clinical practice. These must be determined in each individual patient if required clinically.

The dose estimations shown in Table 5 illustrate the large impact of variance in PK on the calculated dosing needed to maintain any predetermined trough level during prophylactic treatment. They also show that the impact of variance in *t*_½_ is considerable while variance in recovery is of minor importance. The trough (48-h) level is defined by the terminal part of the biphasic curve. Consequently, very similar levels, and thus dose requirements, were estimated when the first part of the curve (including the measured recovery) was ignored.

In summary, comparison of the PK of FVIII between 1–6- and 10–65-year-old hemophilia A patients demonstrated some clear relationships with age. However, reduction of the PK blood sampling schedule could account for up to one half of the observed difference in some key PK parameters. Consequently, differences in blood sampling schedules should be taken into consideration when results from PK studies are compared. However, more important from a dosing point of view is that patient characteristics such as age and ratio weight showed only weak relationships with PK. The unexplained biological variance far exceeded that which could be attributed to known factors. Therefore, PK for dose tailoring cannot be predicted from these characteristics, but must be determined in each individual. The influence of reducing the blood sampling was also minor in comparison to the true variance between patients, both as regards PK parameter values and dose calculations. This suggests that a limited blood sampling protocol can be used in practice. Because intrapatient variability in PK is lower than interpatient variability, the determined PK parameters can then be assumed to be representative for that patient and thus be useful for dose tailoring of FVIII.

## Addendum

All authors contributed to the concept and design of the study, interpretation of data, critical writing or revising the intellectual content, and approval of the final version of this manuscript to be published; a subset of authors (S. Björkman, M. Oh, P. Schroth and S. Fritsch) contributed to the analysis of data.

## References

[1] Ahlberg A (1965). Haemophilia in Sweden VII. Incidence, treatment and prophylaxis of arthropathy and other musculo-skeletal manifestations of haemophilia A and B. Acta Orthop Scand.

[2] Nilsson IM, Berntorp E, Löfqvist T, Pettersson H (1992). Twenty-five years’ experience of prophylactic treatment in severe haemophilia A and B. J Intern Med.

[3] Collins PW, Blanchette VS, Fischer K, Björkman S, Oh M, Fritsch S, Schroth P, Spotts G, Astermark J, Ewenstein B, rAHF-PFM Study Group (2009). Break-through bleeding in relation to predicted factor VIII levels in patients receiving prophylactic treatment for severe haemophilia A. J Thromb Haemost.

[4] Messori A, Longo G, Morfini M, Donati-Cori G, Matucci M, Ruffo S, Tendi E, Vannini S (1984). Individualizing of factor VIII dosage. J Clin Hosp Pharm.

[5] Messori A, Longo G, Matucci M, Morfini M, Rossi Ferrini PL (1987). Clinical pharmacokinetics of factor VIII in patients with classic haemophilia. Clin Pharmacokinet.

[6] Carlsson M, Berntorp E, Björkman S, Lindvall K (1993). Pharmacokinetic dosing in prophylactic treatment of hemophilia A. Eur J Haematol.

[7] Björkman S, Carlsson M (1997). The pharmacokinetics of factor VIII and factor IX: methodology, pitfalls and applications. Haemophilia.

[8] Carlsson M, Berntorp E, Björkman S, Lethagen S, Ljung R (1997). Improved cost-effectiveness by pharmacokinetic dosing of factor VIII in prophylactic treatment of haemophilia A. Haemophilia.

[9] Björkman S, Berntorp E (2001). Pharmacokinetics of coagulation factors: clinical relevance for patients with haemophilia. Clin Pharmacokinet.

[10] Björkman S (2003). Prophylactic dosing of factor VIII and factor IX from a clinical pharmacokinetic perspective. Haemophilia.

[11] Shapiro AD, Korth-Bradley J, Poon MC (2005). Use of pharmacokinetics in the coagulation factor treatment of patients with haemophilia. Haemophilia.

[12] Matucci M, Messori A, Donati-Cori G, Longo G, Vannini S, Morfini M, Tendi E, Rossi-Ferrini PL (1985). Kinetic evaluation of four factor VIII concentrates by model-independent methods. Scand J Haematol.

[13] van Dijk K, van der Bom JG, Lenting PJ, de Groot PG, Mauser-Bunschoten EP, Roosendaal G, Grobbee DE, van den Berg M (2005). Factor VIII half-life and clinical phenotype of severe hemophilia A. Haematologica.

[14] Barnes C, Lillicrap D, Pazmino-Canizares J, Blanchette VS, Stain AM, Clark D, Hensmen C, Carcao M (2006). Pharmacokinetics of recombinant factor VIII (Kogenate-FS) in children and causes of inter-patient pharmacokinetic variability. Haemophilia.

[15] Blanchette VS, Shapiro AD, Liesner RJ, Hernández Navarro F, Warrier I, Schroth PC, Spotts G, Ewenstein BM, for the rAHF-PFM Clinical Study Group (2008). Plasma and albumin-free recombinant factor VIII: pharmacokinetics, efficacy and safety in previously treated pediatric patients. J Thromb Haemost.

[16] Björkman S, Folkesson A, Jönsson S (2009). Pharmacokinetics and dose requirements of factor VIII over the age range of 3–74 years: a population analysis based on 50 patients with long-term prophylactic treatment for haemophilia A. Eur J Clin Pharmacol.

[17] Aronstam A, McLellan DS, Wassef M, Mbatha PS (1982). Effect of height and weight on the in vivo recovery of transfused factor VIII C. J Clin Pathol.

[18] Björkman S, Carlsson M, Berntorp E, Stenberg P (1992). Pharmacokinetics of factor VIII in humans. Obtaining clinically relevant data from comparative studies. Clin Pharmacokinet.

[19] Morfini M, Lee M, Messori A (1991). The design and analysis of half-life and recovery studies for factor VIII and factor IX. Factor VIII/Factor IX Scientific and Standardization Committee of the International Society for Thrombosis and Haemostasis. Thromb Haemost.

[20] Morfini M (2002). Comparative pharmacokinetic studies in haemophilia. Haemophilia.

[21] Lee M, Morfini M, Negrier C, Chamouard V (2006). The pharmacokinetics of coagulation factors. Haemophilia.

[22] Lee M, Morfini M, Schulman S, Ingerslev J, the Factor VIII/Factor IX Scientific and Standardization Committee of the International Society for Thrombosis and Haemostasis The design and analysis of pharmacokinetic studies of coagulation factors. http://www.isth.org/Publications/OfficialCommunications/Factor8and9/Pharmacokinetic.aspx.

[23] Tarantino MD, Collins PW, Hay CR, Shapiro AD, Gruppo RA, Berntorp E, Bray GL, Tonetta SA, Schroth PC, Retzios AD, Rogy SS, Sensel MG, Ewenstein BM, the RAFH-PFM Clinical Study Group (2004). Clinical evaluation of an advanced category antihaemophilic factor prepared using a plasma/albumin-free method: pharmacokinetics, efficacy, and safety in previously treated patients with haemophilia A. Haemophilia.

[24] Lee ML, Poon WY, Kingdon HS (1990). A two-phase linear regression model for biologic half-life data. J Lab Clin Med.

[25] Halls SB About arithmetic formulas for calculating ideal body weight. http://www.halls.md/ideal-weight/devine.htm.

[26] National Center for Health Statistics National Health and Nutrition Examination survey, CDC growth charts: United States. http://www.cdc.gov/growthcharts/.

[27] Fijnvandraat K, Berntorp E, ten Cate JW, Johnsson H, Peters M, Savidge G, Tengborn L, Spira J, Stahl C (1997). Recombinant, B-domain deleted factor VIII (r-VIII SQ): pharmacokinetics and initial safety aspects in hemophilia A patients. Thromb Haemost.

[28] Schwartz RS, Abildgaard CF, Aledort LM, Arkin S, Bloom AL, Brackmann HH, Brettler DB, Fukui H, Hilgartner MW, Inwood MJ, Kasper CK, Kernoff PBA, Levine PH, Lusher JM, Mannucci PM, Scharrer I, MacKenzie MA, Pancham N, Kuo HS, Allred RU, the Recombinant Factor VIII Study Group (1990). Human recombinant DNA-derived antihemophilic factor (factor VIII) in the treatment of hemophilia A. recombinant Factor VIII Study Group. N Engl J Med.

[29] Harrison JF, Bloom AL, Abildgaard CF (1991). The pharmacokinetics of recombinant factor VIII. The rFactor VIII Clinical Trial Group. Semin Hematol.

[30] Björkman S, Folkesson A, Berntorp E (2007). In vivo recovery of factor VIII and factor IX: intra- and interindividual variance in a clinical setting. Haemophilia.

